# A novel fusion protein TBLR1-RARα acts as an oncogene to induce murine promyelocytic leukemia: identification and treatment strategies

**DOI:** 10.1038/s41419-021-03889-0

**Published:** 2021-06-11

**Authors:** Shouyun Li, Xue Yang, Shuang Liu, Yirui Chen, Haiyan Xing, Kejing Tang, Zheng Tian, Yingxi Xu, Qing Rao, Min Wang, Jianxiang Wang

**Affiliations:** 1 State Key Laboratory of Experimental Hematology, Institute of Hematology and Blood Diseases Hospital, Chinese Academy of Medical Sciences and Peking Union Medical College, Tianjin, China; 2 National Clinical Research Center for Blood Diseases, Institute of Hematology and Blood Diseases Hospital, Chinese Academy of Medical Sciences and Peking Union Medical College, Tianjin, China; 3 Present Address: Zhejiang Provincial People’s Hospital, Hangzhou, China

**Keywords:** Acute myeloid leukaemia, Acute myeloid leukaemia

## Abstract

Acute promyelocytic leukemia (APL) is characterized by a specific chromosome translocation involving RARα and its fusion partners. For decades, the advent of all-*trans* retinoic acid (ATRA) synergized with arsenic trioxide (As_2_O_3_) has turned most APL from highly fatal to highly curable. TBLR1-RARα (TR) is the tenth fusion gene of APL identified in our previous study, with its oncogenic role in the pathogenesis of APL not wholly unraveled. In this study, we found the expression of TR in mouse hematopoietic progenitors induces blockade of differentiation with enhanced proliferative capacity in vitro. A novel murine transplantable leukemia model was then established by expressing TR fusion gene in lineage-negative bone marrow mononuclear cells. Characteristics of primary TR mice revealed a rapid onset of aggressive leukemia with bleeding diathesis, which recapitulates human APL more accurately than other models. Despite the in vitro sensitivity to ATRA-induced cell differentiation, neither ATRA monotherapy nor combination with As_2_O_3_ confers survival benefit to TR mice, consistent with poor clinical outcome of APL patients with TR fusion gene. Based on histone deacetylation phenotypes implied by bioinformatic analysis, HDAC inhibitors demonstrated significant survival superiority in the survival of TR mice, yielding insights into clinical efficacy against rare types of APL.

## Introduction

Acute promyelocytic leukemia (APL) is a distinct hematopoietic malignancy accounting for ~10–15% of acute myeloid leukemia (AML) cases [1]. More than 95% of APL cases are driven by the t(15;17)(q22;q21) chromosomal translocation that generates the PML-RARα oncoprotein, eliciting differentiation block at the promyelocytic stage and self-renewal enhancement of myeloid progenitors [2].

However, APL is not solely determined by PML-RARα fusion protein. To date, rare RARα partner genes other than PML have been reported, including *PLZF* [3], *NPM1* [4], *NuMA* [5], *STAT5b* [6], *PRKAR1A* [7], *FIP1L1* [8], *BCOR* [9], *OBFC2A* [10], *TBLR1* [11], *GTF2I* [12], and *FNDC3B* [13]. Among these X-RARα fusion genes, TBLR1-RARα (TR) was the tenth RARα chimera identified in our previous study from three cases of APL with t(3;17) chromosomal translocation [11]. Similar to PML-RARα, TR could self-associate into homodimers and form heterodimers with RXRα. Also, TR exhibited diminished transcriptional activity by recruiting more transcriptional corepressors than RARα. Our previous in vitro studies have verified that pharmacologic doses of all-*trans* retinoic acid (ATRA) would trigger the degradation of TR protein with abrogated homodimerization, incomplete corepressor dissociation, and full differentiation. Nevertheless, in vitro characteristics do not always correlate with the bona fide ability of oncoproteins to initiate disease or block differentiation in vivo [11]. Given the poor clinical outcome of TR patients, further identification of in vivo phenotypes and therapeutic strategies remain to be uncovered. What is the role of TR in leukemogenesis? Would it interfere with the differentiation and proliferative capacity of hematopoietic stem/progenitor cells (HSPCs)? Due to a rarity of clinical cases, preclinical mouse modeling of APL with rare fusion genes may help to lay the groundwork for unraveling the molecular basis of potential treatment toward the disease cure.

In general, four approaches have been utilized to successfully generate APL mouse models as follows: transgenic, knock-in, bone marrow transduction, and xenograft. Human and murine cathepsin G (*CTSG*) and migration inhibitory factor-related protein 8 (*MRP8*, also known as *S100A8*) are the most commonly used promoters to develop APL transgenic mouse models. The CTSG/hMRP8-PML-RARα induced slight alterations in myelopoiesis, developing APL with long latency and incomplete penetrance [14–16]. Later, some groups attempted to generate APL models by transducing murine bone marrow mononuclear cells (BMMNCs) with retroviral vectors harboring the PML-RARα fusion gene. Among them, Minucci et al. [17] reported that inoculation of lineage-negative (lin^−^) cells expressing PML-RARα into irradiated mice propagated promyelocytic leukemia in >80% of recipients at short latency. Apart from diverse models of PML-RARα mentioned above, there were relatively few murine models of rare RARα fusion genes. Although the cathepsin G promoter has also been used to generate transgenic mice expressing PLZF-RARα [18, 19], NPM-RARα [18], and NuMA-RARα [20], myeloid neoplasia generated by these rare RARα fusion genes also differ greatly in disease penetrance and latency. In this study, for the first time, a novel APL mouse model of TR was established by transducing mouse BMMNC lin^−^ cells with retroviral vectors harboring the TR fusion gene. Specific phenotypes mediated by this oncogene were then characterized and several treatment attempts are initiated, which may fill the gap in understanding TR-induced APL and lead to insights into the principles underlying leukemogenesis mediated by rare RARα fusion genes.

Despite the remarkable success of retinoid acid (RA) and arsenic trioxide (As_2_O_3_) combinational treatment over the course of APL research, rare RARα fusion proteins exhibit distinct mechanism and diverse drug responses. For example, those harboring PLZF-RARα and STAT5B-RARα are not sensitive to ATRA therapy [6, 21]. Given the poor outcome of patients in clinical cases, although TR displayed in vitro sensitivity towards ATRA-induced differentiation, whether this therapy leads to cure in vivo remains unknown.

Would TR mice benefit from ATRA or concomitant As_2_O_3_ therapy? If not, how to explain the discrepancy between the in vitro and in vivo studies? Furthermore, is there any other potential treatment option? To address these questions, here we utilized the established new murine model to characterize phenotypes of TR APL through a series of assays and transcriptome analysis. Then, TR mice were challenged with several treatment regimens. Contrary to expectation, standard ATRA and As_2_O_3_ did not bring survival benefit to TR mice. Instead, based on in vitro phenotypes and on potential drug response predicted by transcriptome profile, histone deacetylase inhibitors (HDACIs) including chidamide and a new suberanilohydroxamic acid (SAHA)–bendamustine hybrid NL-101 significantly prolonged the survival of TR mice, providing therapeutic value for improving APL patient outcome with rare RARα fusion genes.

## Materials and methods

### Mice and cell lines

Six- to 8-week-old female and male C57BL/6 mice were purchased from Institute of Laboratory Animal Sciences (CAMS&PUMC, China). Procedures involving animal experiments were approved by the Institutional Animal Care and Use Committee of Peking Union Medical College. HEK293T cells were cultured in Dulbecco’s modified Eagle’s medium with 10% fetal bovine serum (FBS) (Gibco) and were tested free of mycoplasma contamination.

### Plasmid and reagents

Full-length cDNA of TR (GeneBank KF589333) was cloned into pMSCV-IRES-GFP (pMIG) using XholI/EcoRI restriction site with a Flag tag, to yield pMSCV-TR-IRES-GFP. The pMIG plasmid (MSCV-IRES-GFP) was a kind gift from Dr. Michael H. Tomasson’ lab, Washington University School of Medicine. Retrovirus expressing TR or empty vector (vehicle) were used for the following transduction experiment. After transduction, GFP^+^ cells expressing TR were sorted by BD FACS Aria III System and validated by western blotting for the following in vitro and in vivo assays. ATRA was purchased from Sigma-Aldrich and As_2_O_3_ from Harbin Pharmaceutical Co., Ltd. Chidamide was supplied by Shenzhen ChipScreen Biosciences, Ltd, (Shenzhen, China), and suspended in 0.1% sodium carboxyl methylcellulose (CMC-Na). NL-101 was a kind gift from Hangzhou Mingsheng Institute of Pharmaceutical Research (Hangzhou, China).

### Construction of TR murine model and in vivo drug treatment

Lin^−^ cells from C57BL/6 mice bone marrow (BM) were isolated with Lineage Cell Depletion Kit (Miltenyi Biotec) and pre-stimulated for 12 h in Iscove’s modified Dulbecco’s medium (IMDM) containing 20 ng/ml interleukin (IL)-3, 20 ng/ml IL-6, and 50 ng/ml mouse stem cell factor (mSCF), then infected with retrovirus expressing TR or empty vector, respectively. A total of 15 lethally irradiated recipient mice were intravenously transplanted with 5 × 10^5^ GFP^+^ lin^−^ cells and the incidence of leukemia was observed.

Mice used in drug-treatment experiments were non-irradiated or sublethally irradiated recipient mice generated by intravenous inoculation of 1 × 10^6^ GFP^+^ spleen cells of TR leukemia mice. For each experiment, six mice were randomly assigned to each group. The percentage of GFP^+^ cells of peripheral blood and body weight were measured dynamically. The survival time of each group was recorded and compared.

### RNA and protein analysis

Total RNA was isolated and synthesized into cDNA. Primers used for PCR amplification were as follows: (1) TR-F-1: 5′-CCGCTCGAGATGAGTATAAGCAGT GATG-3′ and TR-R-1: 5′-CGGAATTCTCACTTATCGTCGTCATCCTTGTAATCG TTAACCGGGGAGTGGGTGGCCGG-3′. The PCR product is 1685 bp. (2) TR-F-2: 5′-ATGCCGTAATGCCTGATG-3′ and TR-R-2: 5′-GAACTGCTGCTCTGGGTCT-3′. The PCR product is 199 bp. Primers for the candidate genes were searched in PrimerBank (http://pga.mgh.harvard.edu/primerbank/). Western blot experiments were performed for protein analysis using antibody against Flag tag (Sigma-Aldrich, catalog number F1804).

### Ex vivo differentiation and colony formation assay

The transduced GFP^+^ lin^−^ cells were sorted by Arial III flow cytometer (BD Biosciences). For the differentiation study, cells were plated in IMDM containing 15% FBS and cytokines (IL-3, IL-6, and mSCF) with or without ATRA treatment and then collected at different time points for both morphological and surface progenitor/myeloid markers (c-Kit, CD11b, Gr-1, and CD16) analysis. In the colony formation assay, sorted lin^−^ cells were plated in 24-well plates (3000 cells/well) and cultured in M3434 methylcellulose (StemCell Technologies). After 7 days, colonies were counted and the percentage of GFP^+^ cells analyzed by flow cytometry. Then, an equal number of cells were reseeded for serial plating. GFP^+^ colony numbers were calculated as total colony numbers multiplied by GFP^+^ percentage before each plating. Cell colonies were photographed under Operetta CLS High Content Analysis System (Perkinelmer).

### RNA-seq and data analysis

Total RNA was collected from the following samples: purified lin^−^ cells from healthy C57BL/6 mice BMMNCs as control, sorted GFP^+^ cells from BMMNCs of the third generation of TR leukemia mice. RNA samples were sent to Beijing Genomics Institute for library preparation and screening of differentially expressed genes (DEGs). RNA sequencing (RNA-seq) libraries were sequenced via Illumina HiSeqTM 2000. Raw reads are saved as FASTQ file and are filtered into clean reads, and then mapped to genome reference using BWA and Bowtie. Gene expression levels are quantified by RNA-Seq by Expectation-Maximization (RSEM). Screening of DEGs between two samples are based on the analysis method of the poisson distribution. “False discovery rate ≤ 0.001 and the Log2Ratio ≥ 1” are used as the threshold to judge the significance of gene expression difference. Volcano plots were generated using R package ggplot2. Venn diagrams were created with the open webtool Venny (https://bioinfogp.cnb.csic.es/tools/venny/), to depict the overlap between DEGs. Subsequent enrichment analyses of Gene Ontology (GO), Kyoto Encyclopedia of Genes and Genomes (KEGG), and gene set enrichment analysis (GSEA) were performed and visualized using R package clusterprofiler. GO terms were summarized using REVIGO.

### Statistics

GraphPad Prism 6.0 software and SPSS 12.01 software were used for statistical analysis. Statistical comparisons were made using Student’s *t*-test. The survival time was measured by Kaplan–Maier method and the log-rank analysis. Significant differences were indicated with asterisks (*P < 0.05; **P < 0.01; ***P < 0.001).

## Results

### TR blocks myeloid differentiation and increases proliferative capacity of mouse HSPC

**Fig. 1:**
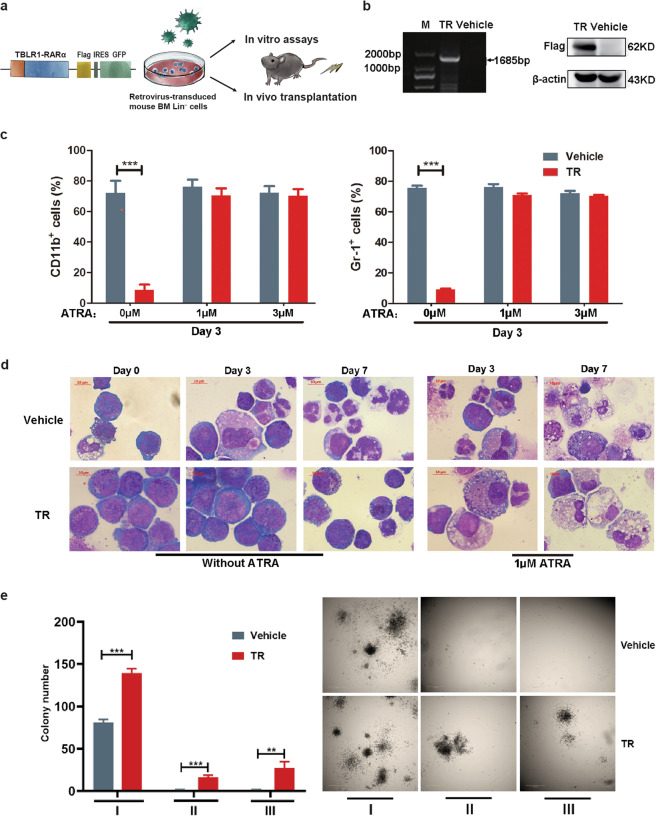
Schematic plot of TR expression vector and in vitro studies. **a** Structure of pMSCV-TBLR1-RARα-flag-IRES-GFP plasmid and construction of murine model based on virus transduction. A Flag tag was inserted in the 3′-end to identify the expression of fusion protein by western blotting. **b** TBLR1-RARα expression detected by western blotting in transduced lin^−^ cells. Lin^−^ cells transduced with retroviruses expressing TBLR1-RARα or empty vector are referred to as “TR” and “vehicle”, respectively. **c**, **d** Analysis and morphology changes (Wright–Giemsa staining; scale bar, 10 μm) of TR and vehicle transduced lin^−^ cells incubated with or without ATRA for 3 or 7 days. **e** Colony-forming ability of TR-transduced lin^−^ cells and vehicles. Serial colony formation assay was performed and the colonies were counted. Mean data and SD (bars) were from triplicate experiments using triplicate samples. Significant differences were indicated with asterisks (*P < 0.05; **P < 0.01; ***P < 0.001).

To determine the influence of TR on HSPCs, the pMSCV-TR-IRES-GFP vector was constructed. Then lin^−^ cells from C57BL/6 mice BMMNCs were enriched and transduced with high-titer retrovirus expressing TR or empty vector. GFP^+^ cells were sorted for the following in vitro assays and in vivo transplantation (Fig. 1a). The expression of fusion gene was confirmed at protein levels (Fig. 1b).

In vitro differentiation assays were carried out with or without ATRA treatment for up to 7 days. As is shown in Fig. 1c, d, after culture for 3 days, a majority of vector-expressing lin^−^ mouse BMMNCs cells were prone to spontaneous differentiation, whereas the expression of TR induced differentiation blockade with only 10% of lin^−^ cells expressing CD11 or Gr-1. With a pharmacological dose of ATRA (1 μM and 3 μM) treatment for 3–7 days, TR-expressing lin^−^ cells could terminally differentiate into mature granulocytes, which again corroborates the in vitro sensitivity of TR APL to ATRA as previously demonstrated in TR-expressing U937 cells [11].

Colony formation assays were then employed to investigate the effects of TR on HSPC self-renewal. It was observed that TR expressing lin^−^ cells produced more colonies in each round of plating compared with that of the control group, which implies the proliferative capacity of TR-expressing lin^−^ cells (Fig. 1e).

### TR acts as an oncogene to induce APL-like disease in mice

**Fig. 2:**
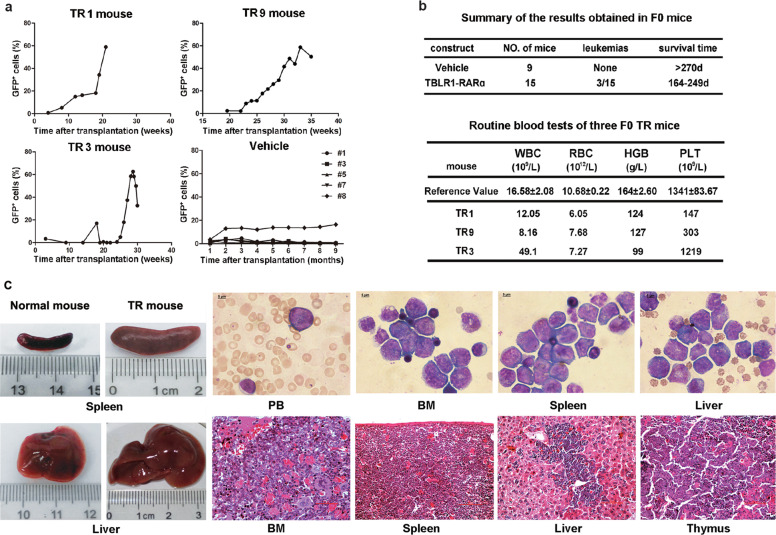
Basic characteristics of primary transplanted mice. **a** Percentage of GFP^+^ cells in peripheral blood in TR mice (TR1, TR3, and TR9 refer to NO.1, NO.3, and NO.9 mouse, respectively) and vehicle groups monitored at each time point. **b** Summary of incidence and survival time of leukemia in primary grafts (upper). Routine blood tests of three primary leukemic mice in the TR group (lower) showed normal WBC counts in TR1 and TR9 mouse with mild anemia and low PLT counts. Data were collected for mice before killing; the reference values were from five wild-type C57BL/6 mice. **c** Left panel: Representative picture of enlarged spleen and liver size from TR mouse (right) compared with control mouse (left). Upper right: immature cells with high nucleus to cytoplasm ratios were observed in the peripheral blood, BM, spleen, and liver by Wright–Giemsa staining (scale bars represent 5 µm). Lower right: histological analysis showed extensive infiltration of primitive myeloid blasts in the bone barrow, spleen, liver, and thymus (HE staining, scale bars: 50 µm in the BM, 100 µm in the spleen, liver, and thymus).

Based on the in vitro results, we hypothesized that TR may act as an oncogene to drive malignant transformation. To investigate whether TR occupied a causal role in APL leukemogenesis, the transplantable leukemia mouse model by TR-expressing retrovirus-infected lin^−^ mice BMMNCs was successfully established. During a 10-month observational period, the circulating GFP^+^ cells in peripheral blood of three mice from the TR group increased gradually over time, compared with that of the vehicle group (Fig. 2a). Finally, 3 out of 15 mice in the TR group developed an APL-like disease, whereas none from the vehicle group died of leukemia (Fig. 2b).

**Fig. 3:**
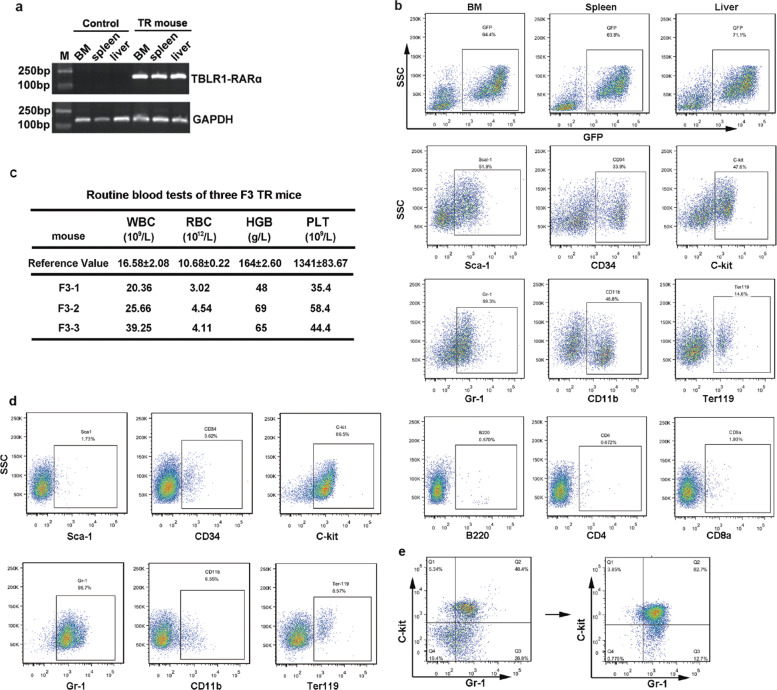
Phenotypic identification of TBLR1-RARα leukemic mice. **a** mRNA analysis showed high expression of TBLR-RARα in different tissues of leukemia mice. **b** BM, spleen, and liver cells from primary TR mice were obtained for surface markers analysis. High percentage of GFP^+^ cells can be detected in different tissues. TR blasts can be defined as Sca-1^+^c-Kit^+^CD34^+^Gr-1^+^CD11b^+^Ter119^+^B220^−^CD4^−^CD8a^−^. **c** Routine blood tests of the third-generation leukemia mice. Data were collected from mice with overt signs of disease; the reference values were from wild-type C57BL/6 mice. **d**, **e** Phenotypes of leukemia cells changed after serial transplantations, with c-Kit^+^Gr-1^+^ cells becoming the main population in the third generation.

All leukemia mice experienced severe body weight loss. Routine blood tests showed mild anemia and thrombocytopenia in NO.1 and NO.9 mice (referred to as TR1 and TR9), with leukocytosis and anemia seen in NO.3 mouse (TR3) (Fig. 2b). Each leukemia mice exhibited massive hepatosplenomegaly (Fig. 2c, left panel). Immature cells were observed in the peripheral blood, BM, spleen, and liver by Wright–Giemsa staining. Histological examination showed the BM, spleen, liver, and thymus infiltrated with primitive myeloid blasts, which confirmed the “leukemic” transformation (Fig. 2c, right panel). TR expression was validated by assessing mRNA using real-time quantitative PCR (Fig. 3a). Flow cytometry analysis of GFP^+^ cells revealed that the progenitor markers Sca-1, CD34, and c-Kit were positive, as well as the myeloid lineage markers Gr-1 and CD11b. A small proportion of GFP^+^ cells were Ter119 positive and almost no cells expressed the lymphatic lineage marker B220, CD3, CD4, and CD8 (Fig. 3b).

Subsequently, spleen cells from primary TR mice were serially transplanted into non-irradiated recipients for two more generations. All recipient mice developed aggressive leukemia with shortened latency of 4 weeks. Among them, the third-generation mice (F3) demonstrated more severe hemogram (leukocytosis, anemia, and thrombocytopenia) compared with primary TR mice (Fig. 3c). Phenotypes of leukemia cells changed after serial transplantations, with c-Kit^+^Gr-1^+^ cells becoming the main population of GFP^+^ cells, accompanied by a reduction of Sca-1^+^, CD34^+^, and CD11b^+^ cells (Fig. 3d, e).

### Different fractions of promyelocytes in TR mice exhibit varying leukemogenic potential

**Fig. 4:**
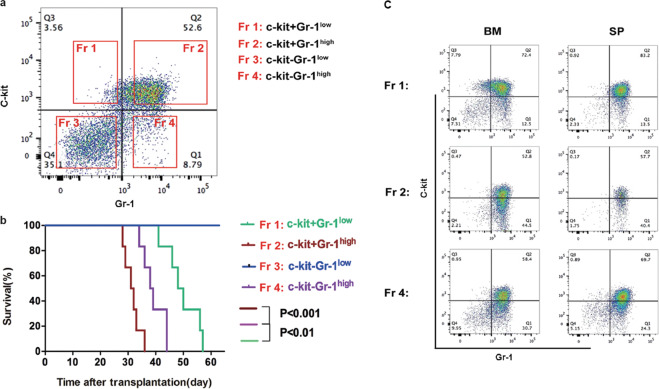
Different fractions of promyelocytes in TR mice exhibit varying leukemogenic potential. **a** Splenic leukemia cells from the third-generation TR mice were divided into four subpopulations according to their expression of cell surface markers C-kit and Gr-1. **b** Kaplan–Meier survival curves of recipient mice transplanted with different cell subpopulations (*n* = 6). **c** Phenotype analysis of recipient mice transplanted with different cell subpopulations.

In Cathepsin-G-PML-RARα knock-in mice, c-Kit^+^Gr-1^+^ cells containing promyelocytes are thought to confer the property of self-renewal and capable of engendering leukemia in secondary recipient mice [22]. To examine the leukemogenic potential of different subpopulations, splenic leukemia cells from the second generation of TR mice were sorted into four fractions according to the expression level of c-kit and Gr-1, namely c-Kit^+^Gr-1^low^ (Fr 1), c-Kit^+^Gr-1^high^ (Fr 2), c-Kit^-^Gr-1^low^ (Fr 3), and c-Kit^-^Gr-1^high^ (Fr 4) (Fig. 4a). Then, 1 × 10^4^ cells from each of the fractions were transplanted into sublethally irradiated recipient mice respectively. As is shown in the survival plot, except for the c-Kit^−^Gr-1^low^ fraction (Fr 3), cells in the other three subpopulations were all able to induce leukemia with the onset of c-Kit^+^Gr-1^high^ fraction (Fr 2) being the fastest (median survival of 31.5 days) and c-Kit^+^Gr-1^low^ fraction (Fr 1) being the slowest (median survival of 49 days) (Fig. 4b). Phenotypic analysis of secondary recipient mice transplanted with three leukemia-engendering fractions revealed that similar to the primary TR mice, most of leukemia cells were c-Kit^+^Gr-1^high^ (Fig. 4c). The long-term repopulating capacity of different fractions was also confirmed by serial transplantation experiments. Meanwhile, c-Kit^+^ leukemia cells were also tested for repopulating capacity in limiting dilution transplantation assay (data not shown).

### Transcriptome profiling associates with leukemic phenotype and predicts drug response

**Fig. 5:**
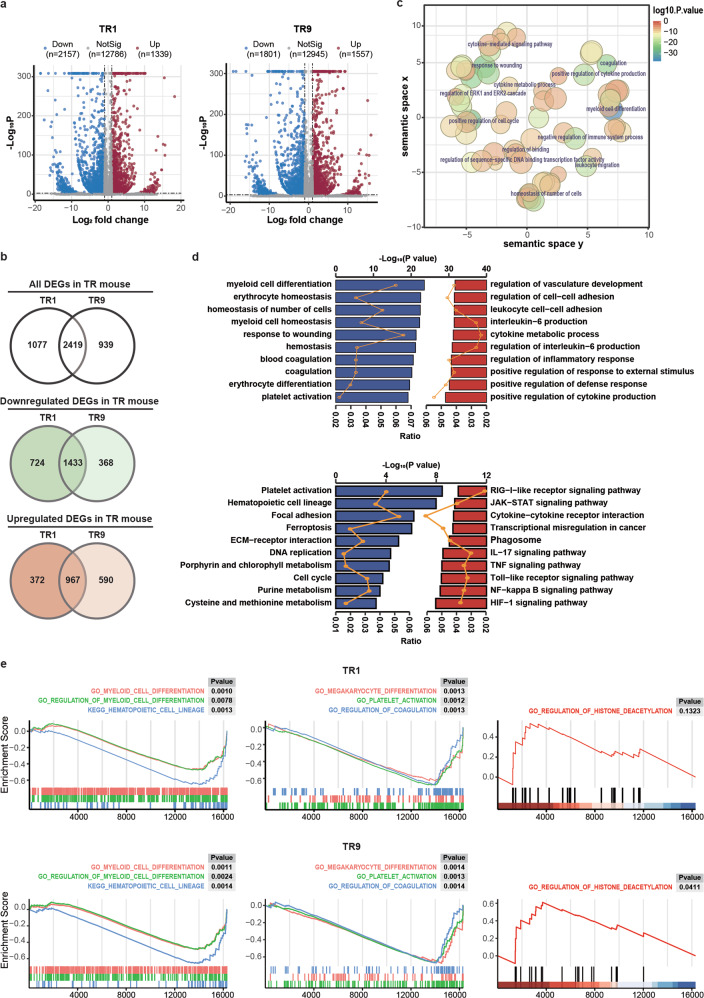
Transcriptional signature of TBLR1-RARα leukemic mice. **a** Volcano plots reflect overall gene expression in TR1 and TR9 mice compared with control mice. Significant upregulated and downregulated DEGs were shown in red and blue, significantly. **b** Venn diagrams show the overlap between DEGs of TR1 and TR9. **c** GO enriched terms among common DEGs were summarized and visualized as a scatter plot using REVIGO. The circle color represents the log10-transformed *p*-value and the circle size represents the number of genes enriched in one term. **d** GO enrichment (upper panel) and KEGG pathway (lower panel) were analyzed by ClusterProfiler. The bar chart represents significance of gene enrichment for GO term or KEGG pathway. The orange lines indicate the percentage of enrichment ratio. **e** Gene set enrichment analysis (GSEA) was performed in DEGs of TR1 and TR9 leukemia mice. Significant gene sets (*p* < 0.05) identified using GSEA include phenotypes of differentiation (left), bleeding diathesis (medium), and histone deacetylation (right).

To decipher the molecular basis of TR-expressing APL, RNA-seq was performed to analyze differential gene expression between GFP^+^ cells of TR mice (TR1 and TR9) and lin^−^ cells of control mice. Volcano plot (Fig. 5a) exhibit the global expression changes of all DEGs, with more downregulated genes than upregulated genes, indicating transcriptional repression mediated by TR fusion gene. Venn diagram shows the overlap between DEGs of TR1 and TR9 (Fig. 5b). Further analyses based on these data were carried out. First, significant GO analysis of all overlapped DEGs is visualized with REVIGO, predominantly enriched in terms related to leukemia phenotypes including myeloid cell differentiation, coagulation, and regulation of immune system process, etc. (Fig. 5c). Then the common downregulated and upregulated genes overlapped in Fig. 5b are mapped to GO (upper panel) and KEGG (lower panel) analysis (Fig. 5d). Consistent with in vitro and in vivo phenotypes, GO analysis showed that downregulated genes are most significantly enriched in myeloid cell differentiation and blood coagulation. Meanwhile, upregulated genes showed enrichment mainly in the regulation of cytokine production, inflammatory response, and cell–cell adhesion. Concerning KEGG pathways, pathways enriched most significantly by upregulated genes involve transcriptional misregulation in cancer and in canonical cancer pathways, including mitogen-activated protein kinase (MAPK) signaling pathway, Janus kinase/signal transducer and activator of transcription (JAK/STAT) signaling pathway, and nuclear factor-κB (NF-κB) signaling pathway, etc. Besides, most prominent pathways enriched in downregulated genes include platelet activation, ferroptosis, extracellular matrix–receptor (ECM–receptor) interaction, and metabolic pathways, etc. We next performed GSEA analysis to verify the enrichment of specific gene sets related to leukemogenesis. As displayed in Fig. 5e, a group of GSEA terms negatively enriched in TR1 and TR9 mice suggested myeloid differentiation block (left) and bleeding diathesis (middle), consistent with in vitro and in vivo phenotypes of TR mouse model. Beyond this, it should be noted that analysis of TR9 mouse revealed an enrichment of histone deacetylation (upper right). Despite the known mechanism of aberrant chromatin acetylation in APL, we did see recruitment of endogenous transcriptional corepressors including HDAC3 in 293T cells expressing TR, which could not completely release from TR/RXR fusion protein with treatment of ATRA [11]. Combining GESA results and validated in vitro studies, potential efficacy of HDACI treatment targeting histone deacetylation underlying TR-driven APL could be predicted.

### HDACIs rather than ATRA or As_2_O_3_ confer survival advantage against TR mice

**Fig. 6:**
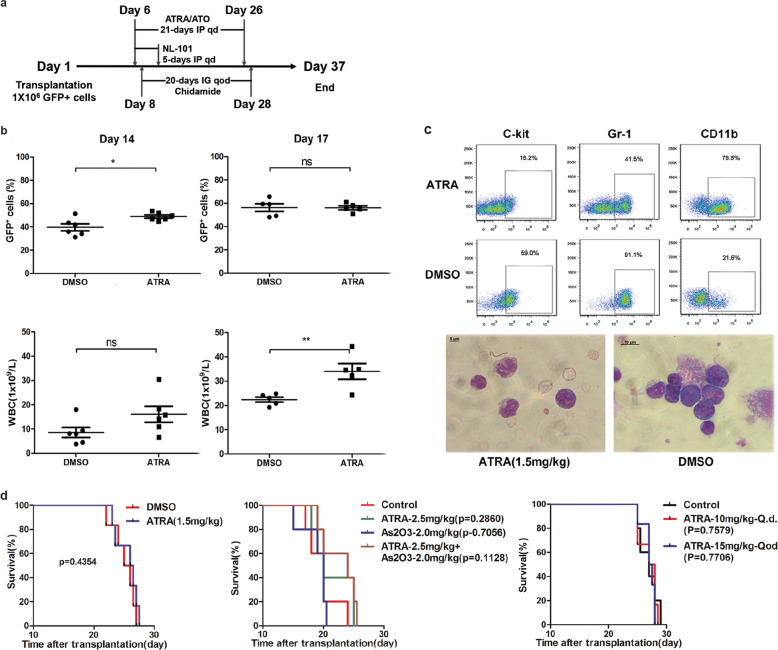
ATRA and As_2_O_3_ failed to prolong the survival of TR mice in vivo. **a** Schematic diagram of overall transplantation and treatment schedule. IP, intraperitoneal administration; IG, intragastrical administration; qd, once daily; qod, once every other day. **b** Sublethally irradiated mice were treated with ATRA (1.5 mg/kg, day 6–26, IP qd) or DMSO (day 6–26, IP, qd) for 5 days after inoculation. The percentages of GFP^+^ cells and WBC counts in the peripheral blood were monitored at day 14 and 17 after the start of treatment. **c** Phenotypes and morphology analysis of GFP^+^ cells from peripheral blood after treatment. **d** Left panel: Kaplan–Meier survival curves of DMSO and ATRA (1.5 mg/kg)-treated groups. Middle panel: survival curves of ATRA and AS_2_O_3_ alone or combination treatment. Right panel: survival curves of the recipients treated with higher doses of ATRA (10 mg/kg, day 6–26, IP, qd; 15 mg/kg, day 6–26, IP, qod) and DMSO (day 6–26, IP, qd).

Although in vitro differentiation assay confirmed the sensitivity of TR-expressing lin^−^ cells to ATRA, the in vivo effectiveness of either monotherapy or combination therapy of ATRA and As_2_O_3_ still awaits further validation. It was observed that cells from TR1 and TR9 mice presented certain differentiated characteristics upon ATRA treatment (Supplementary Fig. 1a) with phenotypic discordance. Except for differed ratio of CD11b^+^ cells in TR1 and TR9 mice, a significant reduction of c-Kit^+^ cells was observed in TR9 rather than in TR1 mouse (Supplementary Fig. 1b). Given that the APL patient harboring TR failed to achieve clinical remission with ATRA and As_2_O_3_ treatment clinically, as well as a potential HDACIs response mentioned above, we then utilized TR murine model to validate responses of TR mouse to ATRA, As_2_O_3_, and HDACIs in vivo (Fig. 6a).

Five days after transplantation, recipient mice were administered with ATRA (1.5 mg/kg) and dimethyl sulfoxide as control, respectively, for 21 days via intraperitoneal injection. As is shown in Fig. 6b, there was no significant difference in the percentage of GFP^+^ cells between two groups, whereas white blood cell (WBC) counts in ATRA-treated group were higher than that of the control group, which may be attributed to the differentiation effect of ATRA. This is indeed the case, as Fig. 6c revealed, most of the GFP^+^ cells in the peripheral blood were differentiated granulocytes in ATRA group, with immature cells in control mice. Low doses of ATRA failed to prolong the survival of TR mice. Similarly, increased doses of ATRA and As_2_O_3_, as well as the standard combination therapy, conferred no obvious survival benefit (Fig. 6d).

**Fig. 7:**
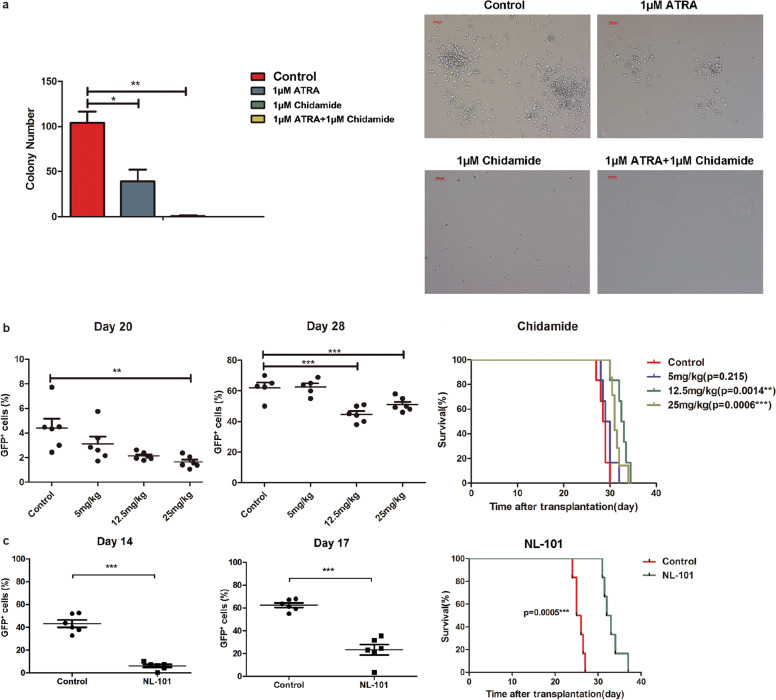
HDACIs confer survival advantage against TR mice. **a** Spleen cells (GFP^+^ cells > 85%) from leukemia mice were plated in methylcellulose medium (M3434) in the presence of 1 µM ATRA and/or 1 µM chidamide, or DMSO solvent for colony formation assay. The colony number and morphology were counted and observed (scale bars, 10 µm). **b** Seven days after transplantation, recipients received different doses of chidamide (5, 12.5, or 25 mg/kg/day, 8–28, IG, qod). Left panel: the percentage of GFP^+^ cells in the peripheral blood of four groups was monitored at day 20 and day 28 after transplantation. Right panel: survival curves of four groups with chidamide or control treatment. **c** Five days after transplantation, recipients were administrated with NL-101 (30 mg/kg, day 6–10, IP, qd). Left panel: the percentage of GFP^+^ cells in the peripheral blood of NL-101 and control groups monitored at day 14 and 17 after therapy initiation. Right panel: survival curves of two groups.

Chidamide, a selective benzamide-type HDACI, has been ratified to treat T-cell lymphoma in China [23]. Previous studies showed that chidamide exerts anti-leukemia effect in hematologic malignancies [24, 25]. Therefore, we first challenged TR mice with chidamide in vitro and in vivo. As shown in Fig. 7a, TR leukemia cells treated with 1 µM ATRA produced fewer colonies than control group, whereas colonies were barely detected when incubated with 1 µM chidamide alone or together with 1 µM ATRA. This result revealed that the proliferative capacity of TR mouse leukemia cells was severely inhibited by chidamide. Consistently, chidamide inhibited the growth of GFP^+^ cells in peripheral blood in a dose-dependent manner in vivo. Leukemic mice receiving 12.5 and 25 mg/kg chidamide survived much longer than mice in control and 5 mg/kg groups (Fig. 7b).

Another novel HDACI NL-101, a compound combining bendamustine with vorinostat (SAHA), was reported to induce apoptosis and DNA damage in myeloid leukemia cells and prolonged the survival of t(8;21) leukemia mice in our previous study [26]. Therefore, the therapeutic effect of NL-101 on TR mice was examined. Intriguingly, GFP^+^ cells dramatically decreased upon NL-101 (30 mg/kg) administration with the median survival time significantly increasing from 25.5 days in the control group to 32.5 days in the NL-101-treated group (Fig. 7c).

## Discussion

Although the paradigmatic combined therapy of ATRA and As_2_O_3_ have benefited most patients with APL to be definitively cured, rare cases of APL with fusion genes other than PML-RARα still exist with molecular pathogenesis remaining largely unclear. In this study, we successfully established a new APL murine model and investigated the in vivo phenotypes and potential therapy for TR-driven APL, which bridges the knowledge gap and provides potential therapeutic strategies for APL with rare fusion genes.

Previous studies have utilized multiple mouse models with regulatory sequences to direct the expression of PML-RARα under promoters of specific stages (*CTSG* and *MRP8*), contributing to antecedent myeloproliferative diseases (MPD) after a long latent period with high WBC counts and different penetrance [14, 15, 18, 20, 27]. The murine transplantable model described in this study using lin^−^ cells expressing TR differs from prior models in several aspects. First, TR leukemia model led to 100% recipient mice developing aggressive leukemia without MPD with an acute onset during serial passaging, which might be partly due to a great increase in the frequency of proper TR targeting. Second, it is known that human APL is characterized by low WBC counts and barely detectable levels of CD34- or CD11b-positive cells [28, 29]. We observed that most primary TR mice presented with normal WBC counts and phenotypes of Sca-1^+^CD34^+^c-Kit^+^Gr-1^+^CD11b^+^, whereas Sca-1^+^, CD34^+^, and CD11b^+^ cells sharply reduced with serial passages. Third, consistent with clinical manifestations of APL patients, TR mice presented with serious hemorrhagic tendency in an advanced disease stage, which were not reported in previous models of PML-RARα [17], PLZF-RARα [18], NPM-RARα [18], and NuMA-RARα [20]. All the above results indicate TR mouse model parallels clinical observations in individuals and recapitulates human APL more accurately than other models. However, in primary graft, there were still 12 mice in the TR group that did not develop leukemia. The compartment where TR is expressed and the specific level of fusion gene expression may have an impact on malignant transformation. Further studies are needed to validate TR mouse models.

Transcriptome profiling of DEGs from TR mouse cells are strongly related to certain leukemic phenotypes. On the one hand, downregulated genes are significantly enriched to GO terms including myeloid cell differentiation and blood coagulation, implying features of differentiation blockage and bleeding diathesis in TR APL, which are also characteristics typical of APL patients with PML-RARα fusion gene. Besides, KEGG enrichment of downregulated genes showed pathways including ferroptosis, ECM–receptor interaction, focal adhesion pathway, and metabolic pathways. Ferroptosis is a specific type of programmed cell death (PCD) and a form of non-apoptotic cell death that depends on iron signaling as well [30]. It has been reported that the resistance to PCD confers drug resistance in AML and the ferroptosis inducer erastin could enhance the sensitivity of AML cells to chemotherapeutic agents [31]. The roles of the ECM–receptor interaction pathway and focal adhesion pathway in AML still remain unclear. In terms of metabolism pathways enriched in downregulated genes, studies have found that leukemia progenitors would activate different programs of the metabolism based on their levels of differentiation blockade [32]. On the other hand, GO and KEGG analysis of upregulated DEGs showed enrichment in regulation of inflammatory response, transcriptional misregulation in cancer, and canonical pathways in AML. For instance, the JAK/STAT pathway is known to be activated by multiple cytokines and cross-talks with MAPK and NF-κB pathways to mediate inflammatory response [33, 34]. Wartman and colleagues used whole genome sequencing to validate the cooperation of JAK1 mutations with PML-RARα, which implicated the JAK/STAT pathway in the pathogenesis of APL [35]. Notably, KEGG pathway of upregulated genes also involved RIG-I-like receptor pathway, whereas RIG-I, namely retinoic acid-inducible gene I, has been reported to upregulate during differentiation of APL induced by RA [36].

Despite the in vitro sensitivity to ATRA-induced cell differentiation, neither single agent of ATRA nor combination with As_2_O_3_ brought survival benefit to TR mice, which is consistent with clinical cases of TR APL. To explain this discrepancy, several aspects should be taken into consideration. For one thing, ATRA-triggered terminal differentiation is insufficient for APL clearance. For another, albeit oncoprotein degradation was responsible for the loss of leukemia-initiating cells (LICs), definitive tumor clearance of APL may be dependent on restoration of PML nuclear bodies (NBs) and subsequent activation of p53 [37]. PLZF-RARα is degraded and yields comparable terminal differentiation compared with PML-RARα upon exposure to ATRA, whereas the clinical outcome of patients did not improve. Similar to PLZF-RARα, TR could be degraded with full differentiation after the treatment of pharmacologic doses of ATRA, whereas both in vivo studies and clinical observations revealed no survival advantage. As PML NBs are not disrupted in PLZF-RARα and TR-driven APL, inactivation of NB-initiated PML-p53 axis may not lead to subsequent abrogating LIC activity. Moreover, when combinational ATRA and As_2_O_3_ allows synergistic degradation, ATRA binds the RARα moiety while As_2_O_3_ alters PML NB biogenesis by direct targeting PML [38]. As_2_O_3_ plays a more profound role in LIC loss and APL eradication than ATRA, by targeting both wild-type PML and fusion genes to restore and promote normal PML NB assembly [39]. Same as the above mentioned, TR would not respond to As_2_O_3_ due to no initial disruption of PML NBs, which may also account for incomplete LIC loss.

Aberrant recruitment of HDACs by fusion oncoproteins was known to play an instrumental role in APL pathogenesis [40], prompting the advance of HDACIs targeting epigenetic alterations to reverse transcriptional repression. The anti-tumor activities of HDACIs mainly involve cellular processes such as apoptosis, cell cycle, and differentiation [41]. For instance, in an ATRA-resistant PLZF-RARα transgenic murine model, SAHA induced apoptosis, growth inhibition, and synergizes with ATRA to increase differentiation. As previously reported, NL-101 has been proved to significantly prolong the survival of t(8;21) leukemia mice with enhanced efficacy than bendamustine [26]. In this study, both chidamide and NL-101 exhibited powerful therapeutic potential for TR mice without obvious toxicity. Apart from well-proven anti-leukemic effects, HDACI was also shown to acetylate p53 and activate transcription [42], based on the context that p53 activation was blunted by PML-RARα expression [43]. As above mentioned, either ATRA or As_2_O_3_-mediated p53 reactivation acts downstream of PML NB restoration. HDACIs might elicit loss of self-renewal in TR-driven APL via other pathways bypassing the PML-p53 axis. However, although HDACIs revealed promising efficacy in TR murine model, which can be performed as a prelude to human trials, there is a current lack of long-term clinical success in HDACI administration [44]. Further investigation in human clinical trials is needed for validation of HDACI efficacy. Future studies of therapeutic regimens could focus on combining ATRA-based differentiation therapy with novel drugs targeting self-renewal pathways, which may mimic and surrogate the effect of As_2_O_3_ in PML-RARα-driven APL to abrogate self-renewal and finally contribute to disease clearance.

In summary, we established and utilized a new APL murine model to identify and characterize in vitro and in vivo phenotypes of TR-induced APL. As opposed to in vitro sensitivity, ATRA and As_2_O_3_ fail to bring survival advantage to TR mice. Instead, HDACIs including chidamide and NL-101 significantly prolonged the survival of TR mice, which may be therapeutically exploited in APL with rare fusion genes.

## Supplementary information

Figure legend of Figure S1

Figure S1

## Data Availability

RNA-seq data have been deposited into the Gene Expression Omnibus (GEO) database under accession number GSE 174247.
