# Supplementary material for: A novel fusion protein TBLR1-RARα acts as an oncogene to induce murine promyelocytic leukemia: identification and treatment strategies
Source: Cell Death Dis. 2021 Jun 11;12(6):607. doi: 10.1038/s41419-021-03889-0 (PMC8196070; doi:10.1038/s41419-021-03889-0)
Supplement: Supplementary file 1 — Figure legend of Figure S1 [file 41419_2021_3889_MOESM1_ESM.docx]

**Supplementary Figure 1: TR murine leukemic cells are sensitive to ATRA in vitro** (a) Morphologic changes of cells from TR1 and TR9 mice treated with different concentrations of ATRA or DMSO solvent as control for 3 days (Wright-Giemsa staining, scale bars represent 10µm). (b) Analysis of cell surface expression of C-kit, CD11b and CD16 incubated in different concentrations of ATRA or DMSO solvent for 3 days.
