# Supplementary figures and images for: A novel fusion protein TBLR1-RARα acts as an oncogene to induce murine promyelocytic leukemia: identification and treatment strategies
Source: Cell Death Dis. 2021 Jun 11;12(6):607. doi: 10.1038/s41419-021-03889-0 (PMC8196070; doi:10.1038/s41419-021-03889-0)

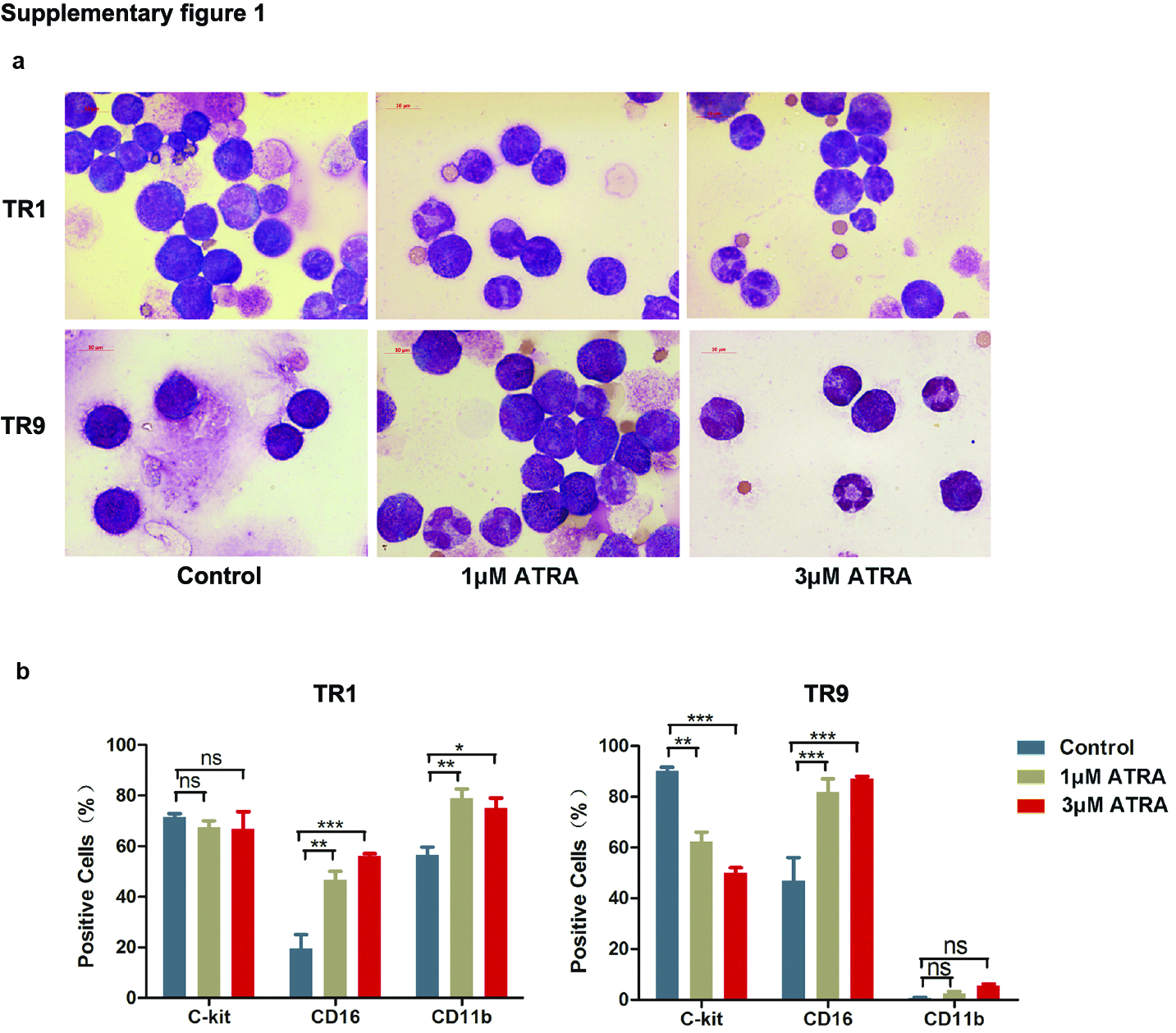

Supplement: Supplementary file 2 — Figure S1 [file 41419_2021_3889_MOESM2_ESM.tif]
